# Calculation of the Electronic Parameters of an Al/DNA/p-Si Schottky Barrier Diode Influenced by Alpha Radiation

**DOI:** 10.3390/s150304810

**Published:** 2015-02-26

**Authors:** Hassan Maktuff Jaber Al-Ta’ii, Yusoff Mohd Amin, Vengadesh Periasamy

**Affiliations:** 1 Department of Physics, Faculty of Science, University of Malaya, 50603 Kuala Lumpur, Malaysia; 2 Department of Physics, Faculty of Science, University of Al-Muthanna, Samawah 66001, Iraq

**Keywords:** alpha particles, Norde’s method, series resistance, height barrier, ideality factor, hypersensitivity

## Abstract

Many types of materials such as inorganic semiconductors have been employed as detectors for nuclear radiation, the importance of which has increased significantly due to recent nuclear catastrophes. Despite the many advantages of this type of materials, the ability to measure direct cellular or biological responses to radiation might improve detector sensitivity. In this context, semiconducting organic materials such as deoxyribonucleic acid or DNA have been studied in recent years. This was established by studying the varying electronic properties of DNA-metal or semiconductor junctions when exposed to radiation. In this work, we investigated the electronics of aluminium (Al)/DNA/silicon (Si) rectifying junctions using their current-voltage (I-V) characteristics when exposed to alpha radiation. Diode parameters such as ideality factor, barrier height and series resistance were determined for different irradiation times. The observed results show significant changes with exposure time or total dosage received. An increased deviation from ideal diode conditions (7.2 to 18.0) was observed when they were bombarded with alpha particles for up to 40 min. Using the conventional technique, barrier height values were observed to generally increase after 2, 6, 10, 20 and 30 min of radiation. The same trend was seen in the values of the series resistance (0.5889–1.423 Ω for 2–8 min). These changes in the electronic properties of the DNA/Si junctions could therefore be utilized in the construction of sensitive alpha particle detectors.

## 1. Introduction

One of the most commonly used rectifying connections in the electronics industry are metal–semiconductor (MS) contact or Schottky barrier diodes (SBDs), employed in a huge number of devices including solar cells, microwave diodes, field-effect photodetectors and transistors (FETs) [1]. These devices have often been used in telecommunication systems, radio astronomy, radar technology, and plasma diagnostics [2]. In recent decades due to the limitations of conventional materials, organic semiconductors such as deoxyribonucleic acid (DNA) and others have found many uses in electrical and optoelectronic applications. DNA can be used in the design and production of novel hybrid semiconductor devices such as photovoltaic devices and diodes [3,4]. Other materials such as conductive polymers and organic compounds have also been shown to achieve rectifying junctions like metal and inorganic semiconductors [5]. 

DNA in particular is a polymorphic molecule which structures are strongly affected by the environment [6,7]. Due to the potential exciting applications of DNA electronics [8], intensified research has been undertaken in the fields of biophysics, chemistry and biomedical research over the past few decades. Khatir *et al*. studied the effect of the magnetic field on Au/DNA/Au diodes. The authors observed a decrease in the barrier height values and increase in the resistance upon an increase in the magnetic field, suggesting potential application as magnetic sensors [9].

Güllü *et al*. studied the changes in electronic properties of Al/DNA/Si sandwich type junctions for current-voltage (I-V) characterization with temperature (200–300K). They determined the effect of temperature on ideality factors, *n*; 1.34 ± 0.02 and 1.70 ± 0.02 at 300 K and 200 K, respectively. The barrier heights (ϕ_b_) were calculated as (0.75 ± 0.01) eV at 300 K, decreasing to (0.61 ± 0.01) eV at 200 K. These values demonstrated the suitability of the Al/DNA/p-type silicon (p-Si) Schottky diode as a good alternative to standard temperature sensors. Meanwhile Okur *et al*. [10] discussed the electrical characterization of Au/DNA/n-Si Schottky diodes by employing the I-V curve and interface state density measurements. Kim *et al*. [11] observed that the conductivity of DNA molecules increased with heat. This was regardless of whether the heating was carried out under ambient or non-ambient conditions, while using N_2_ and O_2_ as the dopant at low temperatures. Furthermore, Gupta *et al*. [12] in their work illustrated that the p-Si/DNA junction can be used as an optical sensor. They measured the photoresponse properties of the diodes, which consist of the ideality factor (1.2 ± 0.1) and barrier height (0.56 ± 0.02 eV). At low radiation frequencies, the capacitance of the diodes increased as a result of the change in the interfacial states. Many researchers in the field of chemistry have studied luminophores and demonstrated a huge difference in oligonucleotide schemes, which have been used to manufacture luminescent DNA-based probes [13].

In our present study of the effect of alpha (α) radiation on DNA, we used a mushroom-based DNA layer on a p-Si wafer to fabricate Al/p-Si/DNA/Al diode structures. To the best of our knowledge, no studies on the effects of alpha radiation on similar structures have ever been reported before. The aim of this study is therefore to fabricate a DNA-based MS diode for potential utilization as an alpha particle detector/sensor. I-V measurements were then performed to analyze the electrical properties of the DNA-based MS diode as the radiation sensitive material. 

## 2. Materials and Methods

### 2.1. Preparation of DNA Solution

A simple preparation procedure of mushroom DNA extracted from fruiting bodies was used for Polymerase Chain Reaction (PCR) amplification. The procedure starts with the collection of minute quantities of mycelium (0.1–1.0 g) from the fruiting body (stipe) of an Oyster mushroom species (*Pleurotus spp*) using sterilized tweezers. Standard procedures [14] were further employed to yield pure DNA samples prior to the PCR process. The DNA of all samples was amplified by PCR (PTC-100TM, MJ Research Inc., Ramsey, MN, USA) using the universal primers ITS1 forward (5'-TCC GTA GGTGA AC CTGCGG-3') and ITS4 reverse (5'-TCCTCCGCTT ATT GATATGC-3'). Amplification reactions were performed in a total volume of 50.0 μL containing 10× PCR buffer 4.0 μL, dNTP mix 2.5 μL, 2.5 μL of each primer, 1.0 μL of Taq polymerase (Cosmo, Seongnam-si, Gyeonggi-do, Korea), 4.0 μL of DNA genomic (Template), and 26.0 μL of sterilized distilled water. PCR amplification as carried-out in 30 cycles at 94 °C for 30 min and denatured at 50 °C for 60 min followed by annealing at 72 °C for an extension of 1 min. Initial denaturing at 95 °C was extended to 5 min and the final extension was at 72 °C for 5 min [15,16]. 

### 2.2. Preparation of Al/DNA/p-Si/Al Junctions 

**Figure 1:**
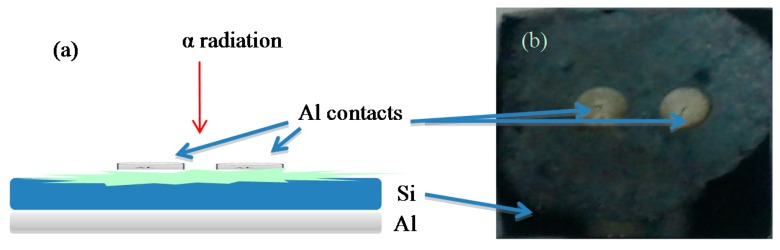
(**a**) Cross section view of Al/DNA/Si surface-type Schottky diodes made on the same substrate and (**b**) the actual images of the sensors.

Junctions were been prepared using a polished p-type Si wafer with [100] orientation with thickness and resistivity of (650 ± 25) µm and (1–10) Ω-cm, respectively (Polishing Corporation of America, Santa Clara, CA, USA). The wafer was chemically cleaned using the RCA cleaning procedure; *i.e.*, 10 min boil in NH_4_ + 6H_2_O + H_2_O_2_ followed by a 10 min boil in HCl + H_2_O_2_ + 6H_2_O solution. Then, a low-resistivity ohmic back contact to the p-type Si wafer was made by using Al, followed by heat treatment at 570 °C for 3 min in N_2_ atmosphere. The native oxide on the front surface of the Si wafer was removed by immersing in HF + H_2_O (1:10) solution before rinsing in deionized water (18.2 MΩ-cm, Barnstead Nanopure II water system, Lake Balboa, CA, USA) for 30 s. Other necessary chemicals (NH_3_, H_2_O_2_, HF, HCl and acetone) were supplied by Sigma Aldrich (St. Louis, MO, USA) and were used without further purification. After which, formation of the organic DNA layer was carried-out by using a micro syringe (Hamilton) containing 10.0 μL DNA with concentration of 1.80 ng/µL from the pre-prepared DNA solution. Schottky metal contacts were then deposited on the organic layer using a metal shadow mask by evaporating Al metal wire (Kurt J. Lesker, Hudson Valley, PA, USA) of 99.999% purity. The Al contacts had dimensions of 2.0 mm, 2000 Å and 3.14 × 10^−2^ cm^2^ of diameter, thickness and area, respectively. All evaporation processes were carried-out in a vacuum thermal metal evaporator coating unit (Edward Auto 306, West Sussex, UK) pressurized to about 10^−7^ mbar. The prepared DNA based devices were air-dried for 24 h in a class 1000 clean room before carrying out the irradiation by alpha particles. Sample irradiation by alpha particles was achieved using ^241^Am with an activity of 150 nCurie and t_1/2_ of 457 years for periods of 2, 4, 6, 8, 10, 20, 30 and 40 min. It’s corresponding I-V profiles were finally recorded in dark using an electrometer (SMU-236, Keithley, OH, USA) at room temperature. Figure 1 depicts the schematic diagram of the DNA based sensors fabricated in this work.

## 3. Results and Discussion

**Figure 2:**
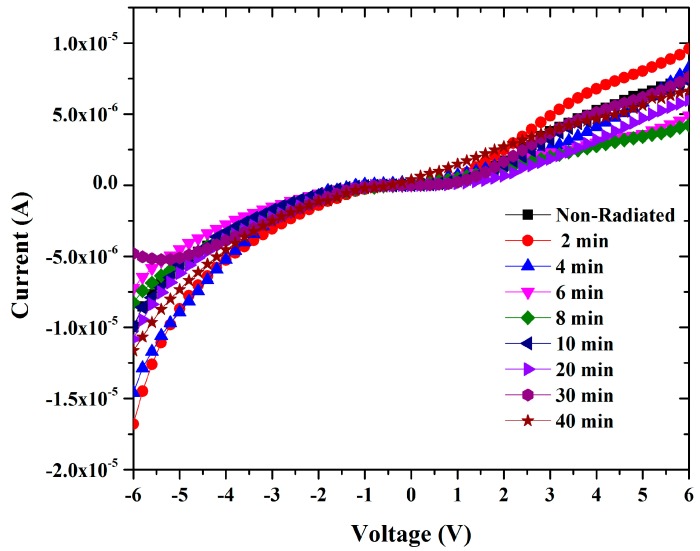
Graphs demonstrates the relationship between current and voltage for forward and reverse biases.

The forward and reverse bias I-V characteristics of the Al/DNA/p-Si/Al junctions at room temperature are given in Figure 2. As can be observed, the I-V characteristics of the device demonstrate a rectifying behavior.

According to the thermionic emission theory, the I-V characteristics of a diode is given by [17];

(1) $$I=I_{0}\exp\left(\frac{qV}{nKT}\right)\left[1-\exp\left(\frac{-qV}{KT}\right)\right]$$

(2) $$I_{0}=AA^{*}T^{2}\left(\frac{-q\Phi }{KT}\right)$$

where q represents the electron charge, the applied voltage by V and effective Richardson constant by symbol A^*^ and equal to 32 A/cm^2^K^2^ for p-type Si [18]. Symbol A meanwhile represents the active diode area, T the absolute temperature, K the Boltzmann constant, n the ideality factor of a SBD and Φ_bo_ the zero bias barrier height. For values of V ˃ 3kT/q, the ideality factor from Equation (1) can be re-written as:

(3) $$n=\frac{q}{KT}\left(\frac{dV}{d\ln I}\right)$$

**Figure 3:**
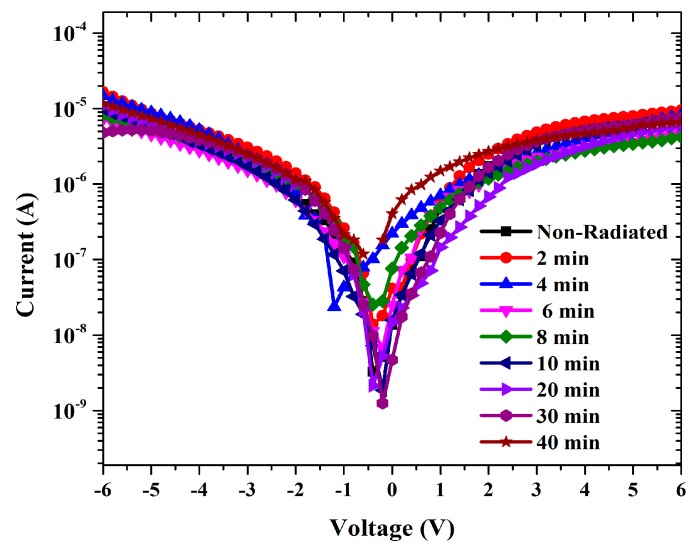
Curves show the I-V characteristics of Al/DNA/p-Si Schottky diode at room temperature.

The ideality factor determined from the slope of the linear region of the forward bias (ln(I)-V) characteristic through the relation in Equation (3) is a measure of conformity of diode to pure thermionic emission [19,20]. Figure 3 shows ideality factor fluctuations of Al/DNA/pi-Si/Al based junctions fabricated in this work calculated using Equation (3).

For both the radiated and non-radiated samples, the linear region of the forward bias I-V plots indicates that the effect of the series resistance in this region is not important. The value of the barrier height (Φ) of the Al/DNA/p-Si/Al Schottky diode was 0.7468 eV before irradiation. The values before and after irradiation (Table 1) were calculated from the y-axis intercepts of the semi log-forward bias I-V plots using Equation (4). It ought to be noted that Φ is the connection potential barrier that exists at the interface between inorganic and organic layers, *i.e.*, at the DNA/p-Si interface:

(4) $$\Phi =\frac{KT}{q}\ln\left(\frac{AA^{*}T^{2}}{I_{0}}\right)$$

**Figure 4:**
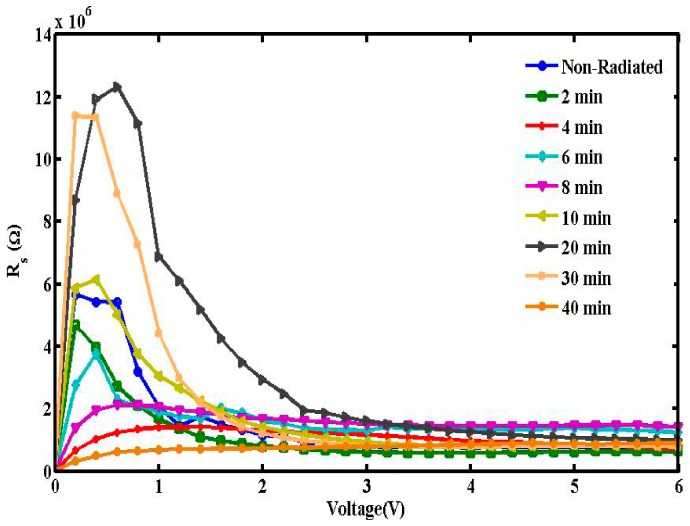
Relation between series resistance and voltage measured using the conventional method.

The values of series resistance are calculated from the junction resistance formula R_S_ = *∂V*/*∂I* from the I-V properties of the diode. The resistance R_S_ versus voltage of the surface-type Schottky diode is demonstrated in Figure 4. From the figure, it can be concluded that at low voltages (≤2.0 V), R_S_ values were the highest for 20, 30 and 10 min in reducing order, followed by the non-radiated sample. However above 2.0 V, the R_S_ values become insignificant. 

**Table 1:** Values of ideality factor, barrier height and series resistance measured.

Radiation Time (Minutes)	Conventional Method			Cheung and Cheung Method				Norde Method		
	n	Φ (eV) (ln(I)-V)	R_S_ (MΩ)	Φ (eV) H(I)	R_S_ (MΩ) H(I)	n dV/lnI	R_S_ (MΩ) dV/lnI	F(V) (V)	Φ (eV) (F-V)	R_S_ (MΩ) (F-V)
0	8.2643	0.7486	0.7772	0.6050	0.81	0.6202	0.066	0.7541	0.7482	0.7342
2	9.0139	0.7553	0.5889	0.6213	0.56	0.2481	0.046	0.7471	0.7612	0.2567
4	7.2683	0.6876	0.7263	0.6742	0.66	1.1628	0.053	0.699	0.6931	0.0861
6	8.5814	0.7429	1.2280	0.6409	1.20	0.2984	0.093	0.7353	0.7294	0.3544
8	12.2826	0.7127	1.4230	0.6758	1.10	1.4341	0.086	0.7180	0.7121	0.1804
10	10.1212	0.7582	0.7985	0.6225	0.84	0.3178	0.067	0.7550	0.7491	0.7601
20	10.9747	0.7594	0.9995	0.6834	0.92	1.3566	0.071	0.7650	0.7591	1.1257
30	7.6935	0.7872	0.7872	0.9878	0.90	0.8915	0.073	0.7720	0.7661	1.4733
40	18.2579	0.6720	0.3217	0.6025	0.76	0.2636	0.060	0.6803	0.6744	0.0416

At high currents, there is always a deviation of the ideality that has been obviously shown to rely on bulk series resistance and the interfacial state density, as one would expect. The lower the series resistance and the interface state density, the better is the range over which lnI(V) does in reality yield a straight line. The Schottky diode factors such as the barrier height Φ_bo_, the series resistance Rs and the ideality factor n were also determined using the technique advanced by Cheung and Cheung [21]. The method’s functions can be written as:

(5) $$\frac{dV}{d\left(\ln I\right)}=IR_{S}+n\frac{KT}{q}$$

(6) $$H\left(I\right)=V-\left(\frac{KT}{q}\right)\ln\left(\frac{I}{AA^{*}T^{2}}\right)$$

therefore:

(7) $$H\left(I\right)=IR_{S}+n\Phi_{b}$$

**Figure 5:**
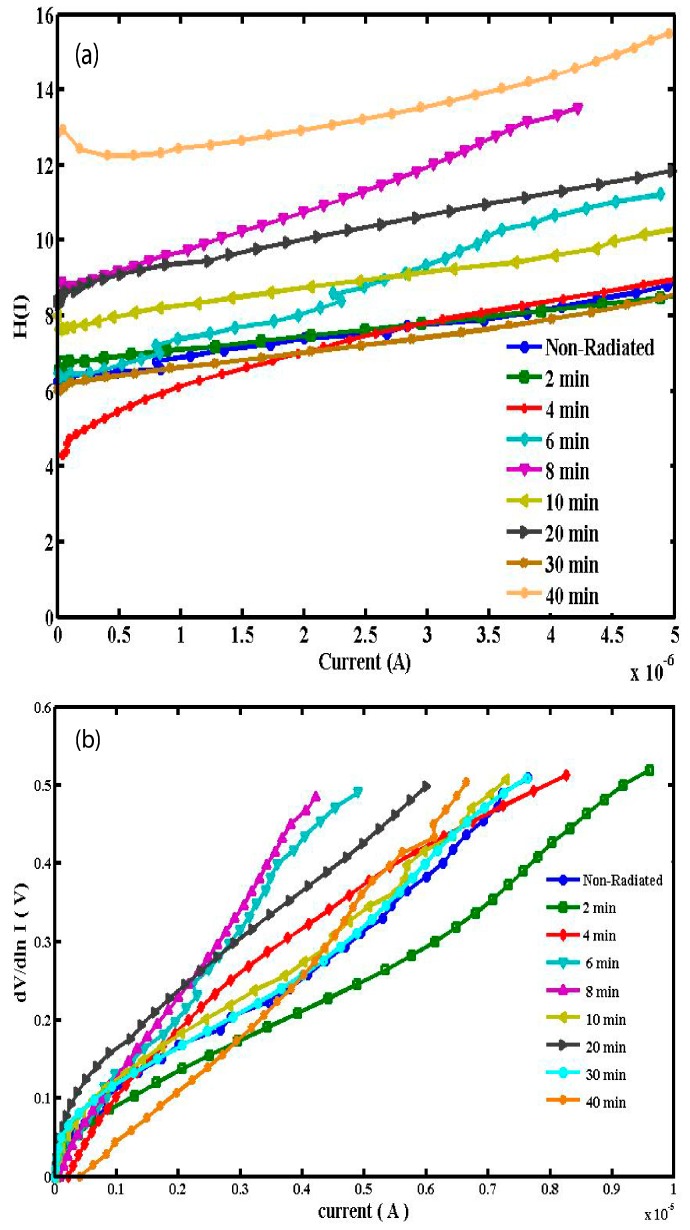
H(I) and dV/d(ln I) *versus* I graphs obtained from forward bias I-V characteristics of Al/DNA/Si/Al Schottky junction diode.

Figure 5a,b shows the experimental H(I) versus I and dV/d(ln I) *versus* I plots for the Al/DNA/Si Schottky diode at room temperature. A plot of H(I) *versus* I (Figure 5a) shows a straight line with intercept at *y*-axis equal to nΦ. Φ was obtained by substituting the n value from Equation (5) and the data of the downward curvature region in the forward bias I-V graph from Equation (7). The slope of this plot also limits R_S_, which can be utilized to check the accuracy of Cheung and Cheung’s method. From H(I) versus I, the Φ and R_S_ values were measured and presented in Table 1. Equation (5) gives a straight line for the data of the downward curvature region in the forward bias I-V graph. 

**Figure 6:**
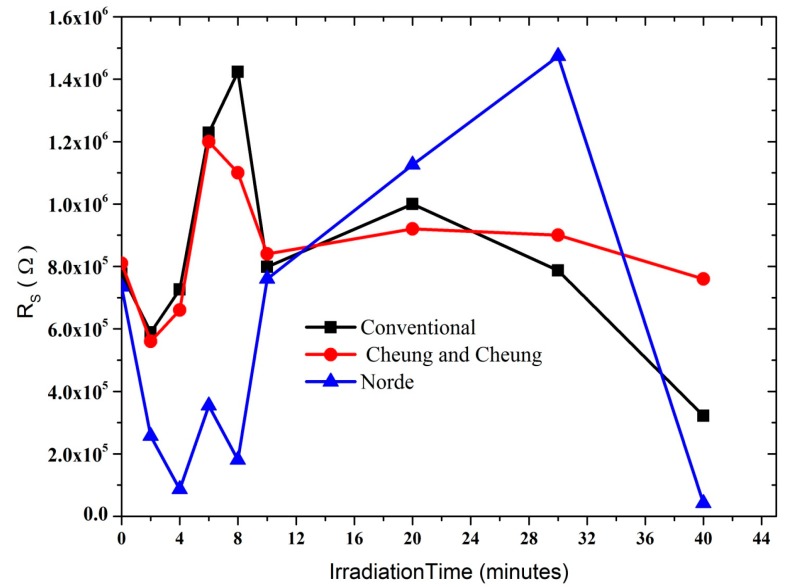
Graphs explaining the relationship between series resistance and alpha radiation time.

**Figure 7:**
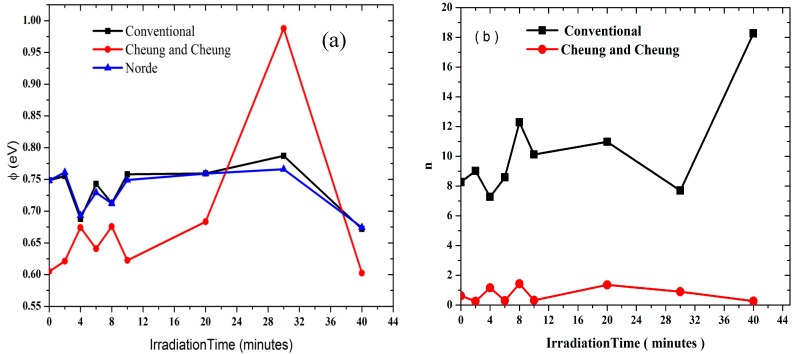
Graphs demonstrating the relationship between ideality factor and barrier height with the radiation time.

Figure 5b shows the plot of dV/d(ln I) *versus* I, from which the values of n and R_S_ were calculated (Table 1). As can be seen in the table, the values of R_S_ obtained from dV/d(ln I) *versus* I and H(I) *versus* I plots are in near agreement with each other. Radiation dose however does play an important role in changing the resistance values, thus the resistance increases gradually at low doses, which therefore enables the DNA to seek self-protection. Plots of Φ, n and R_S_ with radiation periods as shown in Figure 6 and Figure 7 therefore indicate the hypersensitivity phenomena of the DNA at low dose. 

Φ_b_ is the real barrier height derived from the low-voltage part of the forward I-V characteristics. The series resistance was obtained from the straight-line region seen in Figure 5. Using Equation (7), the values of barrier height and the series resistance were then obtained and the results presented in Table 1. The table lists values of n, Φ_b_, R_S_ obtained from both the conventional and the Cheung and Cheung models. Generally, values of n obtained from the dV/d(ln I) versus I curve is lower than that of the forward bias ln I versus V plot. This can be attributed to the effect of the series resistance, interface states and voltage drop across interfacial layers [22,23,24] and radiation effect [25].

Norde’s method is an alternative method to calculate the series resistance and barrier height [26,27]. 

The following function has been derived in the modified Norde method:

(8) $$F\left(V\right)=\frac{V}{\gamma }-\frac{KT}{q}\ln\left(\frac{I}{AA^{*}T^{2}}\right)$$

and effective Schottky barrier height is given by:

(9) $$\Phi =F\left(V_{\min}\right)+\frac{V_{\min}}{\gamma }-\frac{KT}{q}$$

and:

(10) $$R_{S}=\frac{KT}{qI_{0}}$$

where F(V_min_) is the minimum point in the F(V) versus V curve, V_min_ and I_o_ are the corresponding voltage and current respectively. 

**Figure 8:**
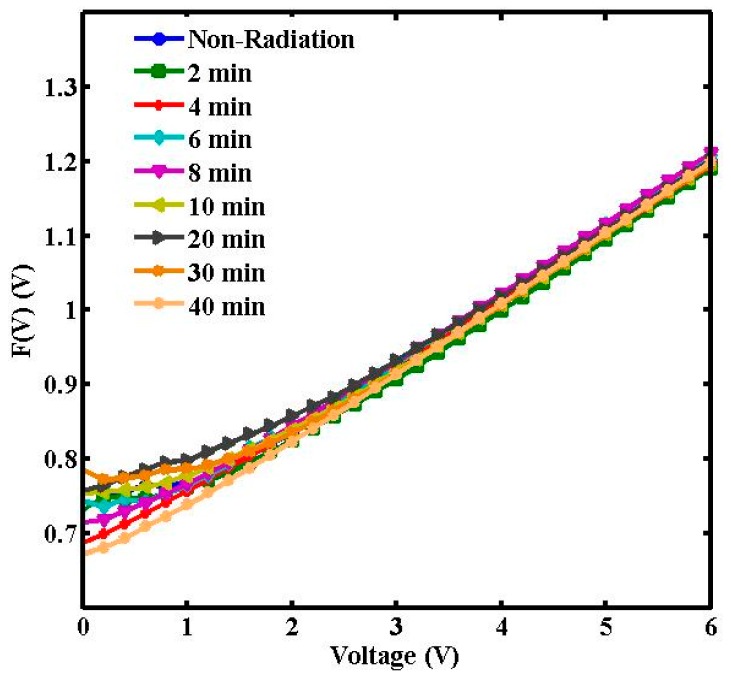
F(V) *vs*. V plots of the radiated and non-radiated Al/DNA/Si Schottky diodes.

A plot of F(V) *versus* V at room temperature is shown in Figure 8. The values of Φ and R_S_ from the plot F(V) *versus* V are listed in Table 1. Norde’s method demonstrates that the values of R_S_ diverge close to non-radiation values when calculated using the conventional method. At lower radiation doses, a significant decrease in series resistance was observed. However, an increase is seen within 10 to 30 min of radiation, registering a drop again at 40 min. The increase may be attributed to the decrease in free carrier concentration and charge mobility. Higher barrier height values denote lower reverse currents [28]. Furthermore, the hypersensitivity phenomenon was responsible for the increase in the Φ and R_S_ values. 

In all the methods, values of the barrier heights were observed to converge. The series resistance initially decreased and later increased with increasing radiation time (Figure 6) as the result of the increasing number of alpha particle tracks. At lower dosages, the ideality factor dropped dramatically demonstrating the hypersensitivity phenomena of the DNA molecules (Figure 7b), which may act to protect against harmful alpha radiation. This phenomenon seems to be similar to the relationship observed between survival curves and radiation dosage [27,29,30]. Schottky barrier height on the other hand has an inverse proportionality relationship with the ideality factor as seen in Figure 7a.

Radiation effect on DNA thin films were also studied using Raman spectroscopic analysis (Renishaw, inVia Raman Microscope, Gloucestershire, UK, 325 and 514 nm lasers) to determine the influence of exposure time. DNA without radiation exposure shows Raman bands of adenine, cytosine, guanine and thymine bases and phosphate backbone groups with different modes of DNA. The Raman bands observed are 1244 cm^−1^ bending of C-H and stretching of C-N bonds; 1418 cm^−1^ stretching bond of adenine; 1290 cm^−1^ C-C bond stretching; 1345 cm^−1^ stretching of C-N and C=C in cytosine; 1576 cm^−1^ C-N-C=C stretching bonds in guanine; 1290 cm^−1^ C-C bond stretching; 1465 cm^−1^ stretching of C-N bonds in thymine; 1068 cm^−1^ symmetric stretching and 1146 cm^−1^ stretching mode of the phosphate backbone. 

**Figure 9:**
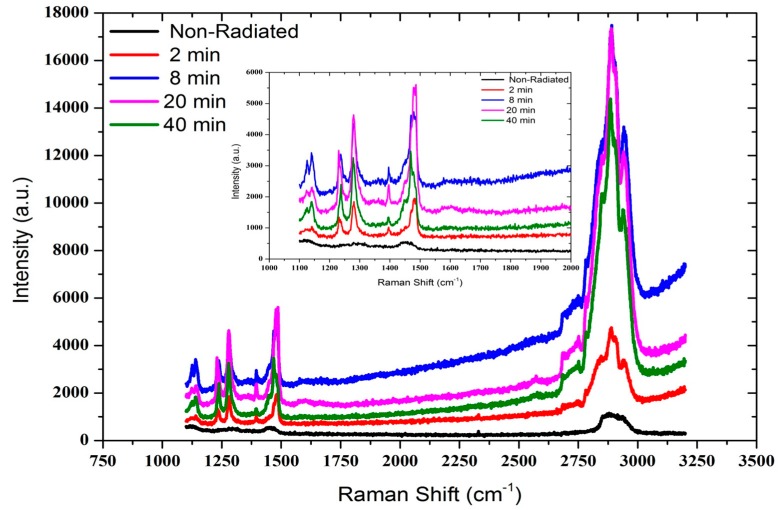
Raman spectra of Al/DNA/p-Si/Al junctions with and without alpha exposure. The insert figure shows the larger view of the peaks to the left of the spectrum.

**Figure 10:**
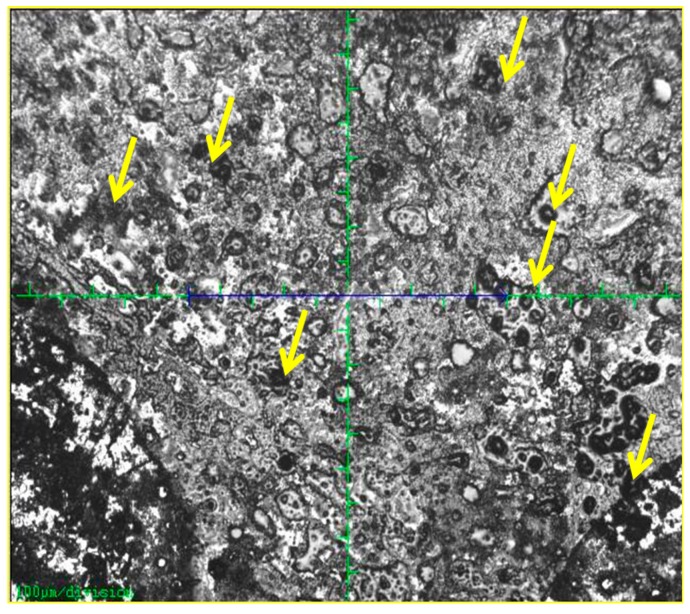
Alpha particle tracks as indicated by the yellow arrows can be clearly observed on the DNA film samples.

Our results are similar with those reported by Kulkarni *et al*. [31]. In the irradiated sample, clear shifting of these peaks can be observed due to the alpha exposures (Figure 9). This is caused by structural damage as a result of absorption of the radiation energy. The peak intensities gradually increased with increasing exposure time (and therefore the dosage) to the ^241^Am radiation source. Increase in the alpha particle irradiation time leads to the linear increase of the number of tracks (Figure 10) and the significant intensity changes observed in the Raman spectrum (Figure 9).

## 4. Conclusions

In this study, we fabricated sandwich type Al/DNA/p-Si Schottky barrier diodes and generated I-V measurements upon exposure to increasing dosage of alpha radiation at room temperature. The values of the series resistances, ideality factors and barrier heights were calculated from the measured non-ideal I-V curve, conventional, Norde and Cheung and Cheung techniques were estimated for the devices. From the conventional method, the calculated Φ value for non-radiated was 0.749 eV, which increased to 0.755 eV after 4 min of radiation. Furthermore barrier height values were observed to increase after 6, 10, 20 and 30 min of radiation, except for 4 and 40 min, which registered a decrease of about 0.68 eV. The electrical resistance increase may be due to drop in the forward current at high voltages whereas the drop in barrier height values is as a result of the growth of the reverse current [28]. Hypersensitivity of DNA to radiation is thought to be responsible for changes in the ideality factors and series resistance. The various parameters studied therefore demonstrate the potential application of the fabricated Al/DNA/p-Si junction type sensor for detecting alpha particles.

## Supplementary Information

Supplementary materials can be accessed at: http://www.mdpi.com/1424-8220/15/3/4810/s1.
